# Ocular Manifestations in Patients with Inflammatory Bowel Disease in the Biologics Era

**DOI:** 10.3390/jcm11154538

**Published:** 2022-08-04

**Authors:** Alix Cuny, Lucas Guillo, Cédric Baumann, Patrick Netter, Silvio Danese, Bénédicte Caron, Laurent Peyrin-Biroulet, Karine Angioi

**Affiliations:** 1 Department of Ophthalmology, Nancy University Hospital, F-54000 Nancy, France; 2 Department of Gastroenterology, University Hospital of Marseille Nord, University of Aix-Marseille, F-13005 Marseille, France; 3 Unit of Methodology, Data Management and Statistic, Nancy University Hospital, F-54000 Nancy, France; 4 Ingénierie Moléculaire et Ingénierie Articulaire (IMoPA), UMR-7365 CNRS, Faculté de Médecine, University Hospital of Nancy, University of Lorraine, F-54000 Vandoeuvre-lès-Nancy, France; 5 Gastroenterology and Endoscopy, IRCCS Ospedale San Raffaele, University Vita-Salute San Raffaele Milano, 20132 Milano, Italy; 6 Department of Gastroenterology, Nancy University Hospital, F-54000 Nancy, France; 7 Délégation à la Recherche Clinique et à l’Innovation, Nancy University Hospital, F-54000 Nancy, France; 8 NGERE (Nutrition-Génétique et Exposition aux Risques Environnementaux), INSERM, University of Lorraine, F-54000 Nancy, France

**Keywords:** extra-intestinal manifestation, inflammatory bowel disease, uveitis, ophthalmology

## Abstract

**Background:** Extra-intestinal manifestations are frequent in inflammatory bowel disease (IBD). Ocular disorders are generally under diagnosed as they are challenging diagnosis. **Aims**: We assessed the prevalence of ophthalmological manifestations in patients with IBD, and investigated characteristics associated with ocular manifestations. **Methods:** We performed a retrospective study including patients followed for IBD and had an ophthalmologic visit from January 2013 to July 2020, among 1432 patients followed during this period. Two groups were considered: the first group included patients whose an ocular diagnosis was considered as “related to IBD”, and the second group including patients whose an ocular diagnosis was considered “not related to IBD”. **Results:** Among 1432 patients with IBD, eighty-seven (6.1%) patients had an ophthalmologic visit. Fifty-three patients (3.7%) were considered to have an ocular extra-intestinal manifestation or an iatrogenic effect of IBD treatment, and 34 diagnoses (2.4%) were considered not related to IBD. Inflammatory surface pathologies were the most frequent (33.2%), including 15 patients with dry eye (17.2%), 9 with blepharitis (10.3%), and 5 with chalazions (meibomian cyst) (5.7%). Uveitis was diagnosed in 13 patients (14.9%), episcleritis in 5 patients (5.7%), and scleritis in 2 patients (2.3%). Characteristics of patients with an ophthalmological diagnosis “related to IBD” versus “not related to IBD” were not statistically different. **Conclusion:** In our cohort, less than 5% of patients had ophthalmological extra-intestinal manifestation. The most frequent ocular diagnosis were dry eye and uveitis. No disease characteristics of IBD were found to be associated with ocular manifestations.

## 1. Introduction

Inflammatory bowel disease (IBD), encompassing ulcerative colitis (UC) and Crohn’s disease (CD), are chronic diseases with a relapsing and remitting course [1,2]. Although the most frequent symptoms involve gastrointestinal tract, extra-intestinal manifestations (EIM) of IBD are common, and affect from 19% to 40% of patients [3,4,5,6]. After rheumatological and dermatological EIMs, ocular disorders are the third most frequent symptoms [3,4]. The main ocular EIM is uveitis, but scleritis, episcleritis and dry eye are also classically described in the literature [3,4,5]. Other rare ocular manifestations were also reported as keratitis (interstitial or ulcerative) [7], optic neuritis, and arterial or venous occlusion [8,9]. Ocular EIMs affected less than 10% of patients with IBD [3,4,8,9,10,11,12]. Although ocular manifestations tend to be varied, non-specific and often mild-to-moderate, they can be serious and lead to blindness [9]. The use of anti-tumor necrosis factor (TNF) agents is well known as the best therapeutic option for ocular EIMs (i.e., adalimumab and infliximab) [13,14,15]. However, although the frequency of ocular inflammatory symptoms has decreased, an increase of iatrogenic damage was observed [16]. There are reports associating TNF inhibitors with the onset or recurrence of inflammatory eye disease consisting of anterior uveitis, posterior uveitis, scleritis, and even orbital myositis [16].

Ocular manifestations are generally under-estimated as they are challenging diagnosis, and need the ophthalmologist’s expertise [8]. Delay in specialized care engages the visual prognosis [17]. To date, risk factors for ocular EIMs in patients with IBD are unknown.

Therefore, we aimed to describe the prevalence of ophthalmological manifestations in a large cohort of patients with IBD, and to identify characteristics that might be associated with the risk of developing ocular EIMs.

## 2. Methods

### 2.1. Study Design and Study Population

We performed a retrospective study conducted at the Nancy University Hospital from January 2013 to July 2020. All adult patients with a confirmed IBD followed in the gastroenterology unit and having performed an ophthalmologic visit at the Nancy University Hospital were eligible to inclusion. IBD diagnosis was based on the combination of clinical symptoms and endoscopic, radiologic, and histological criteria according to the European Crohn’s and Colitis Organization (ECCO) guidelines [18]. This study was approved by the Ethical board of the Nancy University Hospital (2020PI194-135).

### 2.2. Data Collection

We collected the following baseline data for each patient: date of birth, age at ophthalmologic consultation, gender, smoking status (non-smoker, former smoker, or active smoker), date of IBD diagnosis, age at IBD diagnosis, type of IBD, characteristics of IBD according to the Montreal classification [19], date of last visit, activity of IBD according to the Harvey-Bradshaw index (HBI) for CD [20] and partial Mayo score for UC [21] at the time of the last gastroenterological visit, IBD treatment at the time of ophthalmologic consultation, the change of therapy after the ophthalmologic consultation, history of surgery, and history of EIMs or immune-mediated inflammatory diseases (IMIDs) (rheumatological, dermatological or hepatobiliary). For HBI, the disease was considered as “inactive” for a score less than 4, “mild” for 4 to 8, “moderate” for 9 to 12, and “severe” if superior or equal to 12. For partial Mayo score, disease was considered as “inactive” for a score less than or equal to 1, “mild” for 2 to 4, “moderate” for 5 to 6, and “severe” for 7 to 9.

Regarding ophthalmological assessment, we collected reason for outpatient visit, functional and clinical symptoms, ophthalmological diagnosis, supposed link with IBD, treatment, possible complications, and the number of outpatient visits.

### 2.3. Definition of Groups

Concerning the relationship between ophthalmological symptoms and IBD, we considered as “probable” diagnosis for which an association with IBD was known or widely described in the literature (uveitis, episcleritis, scleritis…), “possible” diagnosis included either those less described in the literature, or those also frequent in the general population, or those possibly iatrogenic (dry eye, blepharitis, chalazions…). The diagnoses not described in the literature were classified as “unlikely”, and for the diagnoses that may be related but for which the context did not correspond (for example cataract without advanced age and absence of corticosteroid use, central serous chorioretinopathy and absence of corticosteroid use).

Two groups were considered: the first group included patients whose an ocular diagnosis was considered as “probable” or “possible”, and named “related to IBD”, and the second group including patients whose an ocular diagnosis was considered “unlikely”, and named “not related to IBD”. Switch of IBD treatment was performed in case of failure or intolerance to current therapy, at the discretion of the physician.

### 2.4. Statistical Analyses

Comparative analyses between the two groups (ophthalmic diagnoses “related to IBD” and “not related to IBD”) were made in the total cohort, and according to the IBD type, to try to identify characteristics that might be associated with the risk of developing ocular IEM.

Patient characteristics were described using numbers and percentages. Comparison between groups was done using Fisher’s exact or chi-squared test for categorical variables, and Student or Wilcoxon test for quantitative variables. 

The significance level was set at 0.05. Statistical analyses were performed in collaboration with the Methodology and Statistics Department of the Nancy University Hospital. Analyses was performed using SAS v9.4 (SAS Institute Inc., Cary, NC, USA)

## 3. Results

### 3.1. Patients’ Characteristics 

**Figure 1:**
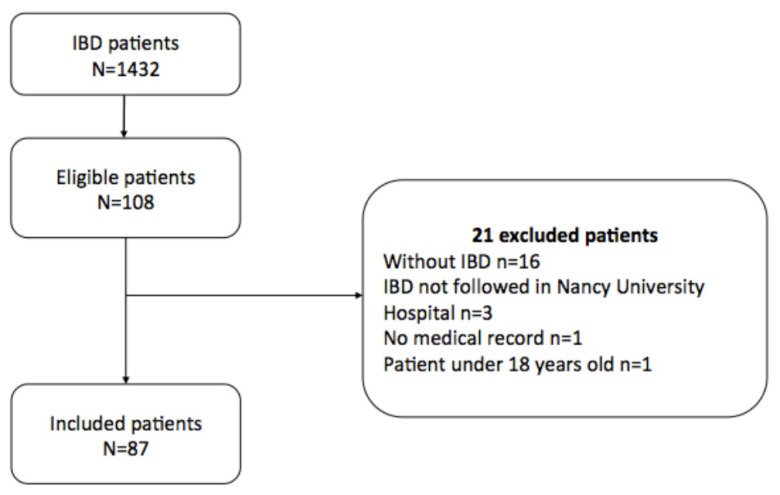
Flow Chart.

A total of 1432 patients with IBD were screened. Among them, 108 had an ophthalmologic visit. Finally, after a carefully review of medical records of these patients, 21 patients were excluded, and a total of 87 patients were included in the study (Figure 1).

Among the 87 included patients, 61 had CD (70.1%) and 26 UC (29.9%) (Table 1). There were 53 women (60.9%). Twenty-six patients were active smokers (29.9%). Median age at diagnosis of IBD was 31 years [21; 43]. Perianal disease concerned 13 patients (21.3%) with CD. Thirty patients (34.5%) needed previous surgery. Seventy patients (80.5%) were treated for their IBD, including anti-TNF (45/87, 51.7%), immunosuppressant (9/87, 10.3%), 5-aminosalicylates (9/87, 10.3%), and other biologics (6/87, 6.9%). Another EIM and/or IMID were reported for 34 patients (39.1%). IBD was inactive in 64 patients (73.6%), 46 patients with CD (75.4%) and 18 patients with UC (69.2%). 

### 3.2. Ocular Symptoms

Eighty-seven patients (6.1%) with IBD performed an ophthalmologic outpatient visit. Fifty-three patients (3.7%) were considered to have an ocular EIM (37/1432) or an iatrogenic effect of IBD treatment (16/1432), and 34 diagnoses (2.4%) were considered not related to IBD.

Median time from IBD diagnosis to the first ophthalmologic outpatient visit was 13 years [8; 22] (Table 2).

A total of 83 patients (95.4%) had an ophthalmologic outpatient visit after the diagnosis of IBD. Ophthalmologic outpatient visit led to the diagnosis of IBD in one patient (1.2%). Two patients (3.4%) were diagnosed after the ophthalmologic outpatient visit. 

The three most frequent symptoms were redness (25%), visual loss (23.2%), and pain (17.2%). Twenty-seven patients complained about visual acuity loss (23.2%).

Twenty-one diagnoses (24.1%) were considered probably related to IBD, 32 (36.8%) possibly related, and 34 (39.1%) unlikely related to IBD (Supplementary Figure S1). Inflammatory surface pathologies were the most frequent (33.2%), including 15 patients with dry eye (17.2%), 9 with blepharitis (10.3%), and 5 with chalazions (meibomian cyst) (5.7%). Uveitis was diagnosed in 13 patients (14.9%), episcleritis in 5 patients (5.7%), and scleritis in 2 patients (2.3%). Twenty-six patients (29.9%) received topical steroid or non-steroidal anti-inflammatory treatment following the ophthalmology consultation. Thirteen patients (14.9%) required a change in their IBD treatment following the ophthalmologic consultation. Six patients (6.9%) had an indication to initiate treatment, and 2 (2.3%) to add systemic corticosteroids. Others were patients on anti-TNF who required a switch to another biologic (*n* = 2), a switch to 5-amino salicylates (*n* = 1), a switch to another anti-TNF (*n* = 1), or a discontinuation of anti-TNF without therapeutic relay (*n* = 1).

Fifteen patients had a diagnosis of dry eye. The main related symptoms were discomfort and eye burns. More than half (*n* = 9) had a superficial punctate keratitis, responsible for pain, visual blur and/or photophobia. 

Thirteen uveitis cases were diagnosed. Twelve uveitis were anterior and one was a pan uveitis. Four were bilateral (*n* = 4). All cases were treated with topical corticosteroids, three of them requiring the use of oral corticosteroids. Two cases were complicated. IBD was inactive in 8 patients (61.5%), and low or moderate in the others. Ten patients had not treatment for IBD. Pan uveitis occurred with anti TNF treatment. Most patients had previous diagnoses of IBD before uveitis (*n* = 9). Uveitis diagnosis was prior to IBD in 3 cases, and concomitant in 1 case. Eight patients had associated arthralgia (with or without ankylosing spondylitis). 

Five episcleritis and 2 scleritis were diagnosed, all were unilateral. IBD was inactive for 4 episcleritis, and one had low activity. All five patients had IBD treatment. One case of scleritis had severe activity of IBD while the other had low activity of IBD. Both patients did not have any IBD treatment. Episcleritis was treated with local anti-inflammatory, and scleritis with general corticotherapy.

### 3.3. Risk Factors for Ocular Manifestations

In this cohort, there was no significant difference between the two groups according to the supposed link with the diagnosis. There was no significant difference for the presence of previous surgery, number of surgeries, use of IBD treatment, and the duration of IBD. There was no significant difference for IBD activity, the presence of another EIM or IMID, and smoking status (Table 3).

In this cohort, switch of IBD treatment was significant for patients with diagnoses “related to IBD” group (*p* = 0.0012).

In patients with CD, 35 patients had an ophthalmologic diagnosis of related to IBD. There was no significant difference between the two groups according to the supposed link with the diagnosis. There was no significant difference for the presence of previous surgery, number of surgeries, use of IBD therapy, phenotype, and clinical activity of CD (Table 4). Switch of IBD treatment was significant for patients with diagnoses “related to IBD” for patients with CD (*p* = 0.0016).

In patients with UC, 18 patients had an ophthalmologic diagnosis of related to IBD. There was no significant difference between the two groups according to the supposed link with the diagnosis. (*p* > 0.05) There was no significant difference for the presence of previous surgery, number of surgeries, presence of IBD therapy, phenotype, and clinical activity of UC (*p* > 0.05) (Table 5). 

## 4. Discussion

Overall, in the cohort of 1432 patients, 87 patients with IBD performed an ophthalmologic outpatient visit. Among them, 53 patients were considered to have an ocular EIM or an iatrogenic effect of IBD treatment.

The prevalence of ocular EIMs in this study was 2.6% (37/1432), in accordance with literature (2–10%) [8,11,12]. Among the 53 patients with diagnoses “related to IBD”, 37 patients were considered to have an ocular EIM. In fact, 37 diagnoses seem to be obviously related to IBD, and 16 had a potential iatrogenic effect (9 blepharitis, 5 chalazions, 1 herpetic keratitis and 1 corneal abscess). In patients with CD, 35 ophthalmological diagnoses were considered related to IBD, with 26 ocular EIM, and 9 potential iatrogenic effects. Other EIMs represent 42.6% of patients with CD. In patients with UC, 18 were considered related to IBD, with 13 ocular EIM, and 5 potential iatrogenic effects. Ocular EIMs represent 50% of patients with UC, among patients with IBD who had an ophthalmologic outpatient visit. Although some authors demonstrated a greater frequency of ocular EIMs in CD versus UC [9,22], it is controversial for others [23,24,25]. Mendoza et al. didn’t found differences between UC and CD [26].

Dry eye were numerous with 15 cases; it represents only 1% of patients with IBD in our study. Lee et al. considered dry eye as the first ocular EIM, with 57% of patients with IBD [27]. However, this was a prospective study in which each IBD patient received an ophthalmologic examination. Dry eye symptoms are very subjective and diagnosis can be made clinically by an ophthalmologist even the absence of symptoms. A large number of our patients suffering of dry eye did not require a consultation at the university hospital and had been treated elsewhere (private ophthalmologist, general practitioner, non-prescription medication). Although frequent in the general population (3.8% to 93% [28,29]), dry eye may be directly related to digestive disease (systemic inflammation inducing hypo secretion [7], hypovitaminosis A [11] or iatrogenic with salicylates [30,31]. Contradictory data exists in the literature regarding anti-TNF use and dry eye [32,33].

Meibomian gland dysfunction concerned 14 patients (9 blepharitis and 5 chalazions). Although frequent in general population (8.8%) [34], blepharitis, and chalazions, are known as adverse effects of anti-TNF use [16]. Contradictory data also existing concerning blepharitis. There are considered as ocular EIMs for some [30,35], and coincident for others [36].

Thirteen uveitis cases were diagnosed in our study. This represented 0.9% of patients with IBD, which is similar to the data of Troncoso et al. (0.5 to 3%) [9]. This is higher than the prevalence in the general population (0.04 to 0.2% in industrialized countries [37,38,39]). As described in the literature, uveitis were possibly bilateral (*n* = 4) [9]. IBD was inactive in the majority of cases. Uveitis does not seem to be related to IBD’s activity, concurring with Troncoso et al. [9]. Ten patients had not treatment for IBD. This is in favor of a protective anti-inflammatory effect of IBD treatments, in particular anti-TNF therapy as described by Susanna et al. [16]. Pan uveitis occurred in a patient with anti TNF treatment, demonstrating the possibility of severe iatrogenic uveitis as described by Toussirot et al. [40]. Most patients had previous diagnoses of IBD before uveitis (*n* = 9). Uveitis diagnosis was prior to IBD in 3 cases, and concomitant in 1 case, in agreement with Zippi et al. [4]. Eight patients had associated arthralgias (with or without ankylosing spondylitis). This is in accordance with Mintz et al. which described more frequent ocular manifestations in patients with arthralgias [8].

Five episcleritis and two scleritis were diagnosed. In our study, prevalence was 5.7% for episcleritis, and 2.3% for scleritis. It is in accordance with the literature for episcleritis (0.2–7.5% [24,25,30,35,36,41,42] but not for scleritis (around 1% [24,43]). This is probably due to the limited data in the literature and small population series. It is more than general population, with prevalence around 0.005% for episcleritis, and 0.0025% for scleritis. IBD was inactive for the majority of patients with episcleritis, no consistent with literature [9,44]. One case of scleritis had severe activity of IBD, and the other had mild activity of IBD. This is consistent with the literature, which describes a frequent correlation with IBD activity [8]. Serious ocular complications can occur in scleritis. Evolution was favorable and no complications were found in our patients, probably because of the rapid management with adapted treatment.

The risk factors associated with the development of ocular EIMs are poorly defined. In our study, only switch of IBD treatment was significant for patients with diagnoses “related to IBD” group, for the total cohort and patients with CD. It was not significant in patients with UC because of small numbers of patients. This highlights the need for optimal control of digestive disease and/or potential iatrogenicity of treatments.

In our study, the majority of patients with uveitis had articular EIM or IMID (61.5%), in accordance with Mintz et al. [8]. Patients with one extra-intestinal manifestation seem to have greater risk to develop another [3]. In the literature, positive association with the presence of joint and/or skin manifestations was found for both UC and CD [12] and with female gender [22,45]. We found this trend for others EIMs, IMIDs, and female gender. (Table 3) There is probably a genetic factor in the development of ocular EIMs. Lin et al. [46] showed a positive association between family history of IBD and ocular EIMs. Orchard et al. [22] identified an association between the development of ocular EIMs and the major histocompatibility complex (HLA). These data were not considered in our study due to a lack of data.

This study was performed on a large patient population in a reference center for IBD management and over a period of several years. This work could serve as a basis for other studies, allowing the calculation of the number of patients necessary to obtain sufficient statistical power.

Limitations of our study include its monocentric and retrospective nature. We couldn’t realize the study over a longer period because computerization of medical records has only been generalized since 2013 at the Nancy University Hospital. Missing data would be expected if we had included records before 2013. Moreover, ophthalmologists in other centers can manage common ophthalmological pathologies in IBD.

Another one limit of this study is the subjective nature of some data. The supposed link between ophthalmologic diagnosis and IBD may thus be subjective in some cases. Dryness is considered as coincidental for some, and as the first EIM for others [30,47]. Furthermore, the association between CSCR and IBD is mentioned in the literature, but usually in a context of corticosteroid use [48,49,50]. So, we have decided to classify CSCR in the diagnoses not related to IBD, because an absence of corticosteroid use in this case.

In conclusion, ocular manifestations associated with IBD remain rare events, although more frequent than in the general population. There mainly include inflammatory ocular entities (uveitis, episcleritis, scleritis, dry eye, blepharitis, chalazion), which may potentially be iatrogenic. No predictive factor to develop ocular EIMs was identified in our cohort. Ocular EIMs are rare but can be severe. A delay in appropriate management can markedly affect the visual prognosis of patients. Generally frequent and less serious pathologies (dry eye, blepharitis with anti-TNF use) can significantly alter patients’ quality of life. Thus, an adapted and early treatment and/or systemic therapeutic modification can help relieve these patients, and avoid ocular complications.

## Figures and Tables

**Table 1:** Characteristics of patients.

Characteristics	Total *n* (%)	CD *n* (%)	UC *n* (%)	*p* *
**Total number of patients**	87	61 (70.1)	26 (29.9)	
**Gender**				0.6871
Female	53 (60.9)	38 (62.3)	15 (57.7)	
Male	34 (39.1)	23 (37.7)	11 (42.3)	
**Smoking status**				0.3651
Active smoker	26 (29.9)	20 (32.8)	6 (23.1)	
Non smoker or former smoker	61 (70.1)	41 (67.2)	20 (76.9)	
**Median age at IBD diagnosis (years [Q1; Q3])**	31 [21; 43]	34 [20; 42]	30 [23; 43]	0.9926
**Montreal classification E (UC)**				
E1 (rectitis)	-	-	9 (34.6)	
E2 (left-sided colitis)	-	-	7 (26.9)	
E3 (extensive colitis)	-	-	10 (38.5)	
**Montreal classification L (CD)**				
L1 (ileitis)	-	18 (29.5)	-	
L2 (colitis)	-	15 (24.6)	-	
L3 (ileocolitis)	-	28 (45.9)	-	
L4 (upper localization)	-	1 (1.6)	-	
**Montreal classification B (CD)**				
B1 (non-stricturing and non-penetrating)	-	41 (67.2)	-	
B2 (stricturing)	-	10 (16.4)	-	
B3 (penetrating)	-	10 (16.4)	-	
**Perianal disease (CD)**	-	13 (21.3)	-	
**Surgery for IBD**	30 (34.5)	28 (45.9)	2 (7.7)	0.0006
0 intervention	57 (65.5)	33 (54.1)	9 (34.6)	
1 intervention	15 (17.2)	14 (23.0)	1 (3.8)	
≥2 interventions	15 (17.2)	14 (23.0)	1 (3.8)	
**IBD treatments**				
Steroids	1 (2.9)	1 (1.6)	0	NR
5-aminosalicylates	9 (19.1)	2 (3.2)	7	NR
Immunosuppressants	9 (19.1)	8 (13.1)	1 (3.8)	NR
Other biologics (vedolizumab, ustekinumab)	6 (15.6)	3 (4.9)	3 (11.5)	NR
Anti TNF (infliximab, adalimumab)	45 (51.7)	33 (54.1)	12 (46.2)	0.4973
**Other EIM**	34 (39.1)	26 (42.6)	8 (30.8)	0.2996
**IMIDs (APS, RA, JA and/or Psoriasis)**	34 (39.1)	27 (44.3)	7 (26.9)	0.1292
**IBD Activity at ophthalmologic visit**			0.9064	
Active	23 (26.4)	15 (24.6)	8 (30.8)	
Inactive	64 (73.6)	46 (75.4)	18 (69.2)	

* Chi-2 or Fisher’s exact test for qualitative variables, test from a Student or Wilcoxon test for quantitative variables. NR: not feasible due to low numbers in one of the modalities (less than 5). APS: ankylosing spondylitis; CD: Crohn’s disease; EIM(s): extra-intestinal manifestation(s); HBI: Harvey-Bradshaw index; IBD: inflammatory bowel disease; IMID(s): immune-mediated inflammatory disease(s); JA: juvenile arthritis; RA: rheumatoid arthritis; TNF: tumor necrosis factor; UC: ulcerative colitis.

**Table 2:** Characteristics of ophthalmological evaluation.

Characteristics	Total *n* (%)
**Median age at ophthalmologic outpatient visit (years [Q1; Q3])**	47 [35; 56]
**Median time from IBD diagnosis to ophthalmologic outpatient visit (years [Q1; Q3])**	13 [8; 22]
**Date of ophthalmologic outpatient visit in comparison with IBD diagnosis**	
Before	3 (3.4)
After	83 (95.4)
Simultaneous	1 (1.2)
**Number of visit**	
1	73 (83.9)
≥2	14 (16.1)
**Reasons for visit**	
Redness	29 (25)
Pain	20 (17.2)
Visual acuity loss	27 (23.2)
Others *	40 (34.4)
**Diagnosis**	
Dry eyes	15 (17.2)
Uveitis	13 (1.9)
Episcleritis	5 (5.7)
Scleritis	2 (2.3)
PUK	1 (1.1)
Blepharitis	9 (10.3)
Chalazion	5 (5.7)
Herpetic keratitis	1 (1.1)
Corneal abscess	1 (1.1)
CRAO	1 (1.1)
Cataract	9 (10.3)
Refractive disorder	8 (9.2)
Systematic control	5 (5.7)
AMD	1 (1.1)
Conjunctivitis	4 (4.6)
Subconjunctival hemorrhage	1 (1.1)
Post-traumatic ulcer	1 (1.1)
CSCR	1 (1.1)
Ophthalmic migraine	1 (1.1)
Valsalva retinopathy	1 (1.1)
Floating bodies	1 (1.1)
**Relationship with IBD**	
Probable	21 (24.1)
Possible	32 (36.8)
Unlikely	34 (39.1)
**Ophthalmological treatment**	
Artificial tears	32 (36.8)
Anti inflammatory (Steroid and non steroid, topic or systemic)	26 (29.9)
Others (anti viral, eyes drops antiseptic, antibiotic)	19 (21.8)
**Ophthalmologic complications**	3 (3.4)
**Switch of IBD treatment**	13 (14.9)

* Others reasons for visit were palpebral edema (*n* = 6), discomfort (*n* = 7), eye burns (*n* = 13), photophobia (*n* = 1), visual blur (*n* = 4), purulent secretions (*n* = 2), tearing (*n* = 3), systematic control (*n* = 2) and myodesopsia (*n* = 2). AMD: age-related macular degeneration; CRAO: central retinal artery occlusion; CRSC: central serous chroioretinopathy; EIM(s): extra-intestinal manifestation(s); IBD: inflammatory bowel disease; IMID(s): immune-mediated inflammatory disease(s); PUK: peripheral ulcerative keratitis.

**Table 3:** Risk factors for ocular manifestations.

	Related to IBD		Not Related to IBD		
	*n* = 53 (60.9%)		*n* = 34 (39.1%)		
Characteristics	*n*	%	*n*	%	*p* *
**Gender**					0.4406
Male	19	35.8	15	44.1	
Female	34	64.2	19	55.9	
**Smoking**					0.0654
Active smoker	12	22.6	14	41.2	
Non-smoker or former smoker	41	77.4	20	58.8	
**Median age at IBD diagnosis (years [Q1; Q3])**	53	34 [23; 42]	34	26.5 [20; 46]	0.6334
**Median age at ophthalmologic outpatient visit (years [Q1; Q3])**	53	47 [35; 44]	34	47.5 [36; 49]	0.401
**Date of ophthalmologic outpatient visit in comparison with IBD diagnosis**					0.3843
Before	3	5.7	0	0	
After	49	92.5	34	100	
Concomitant	1	1.9	0	0	
**Median time from IBD diagnosis to ophthalmologic outpatient visit (years)**					0.7966
<11 years	25	47.2	17	50	
Equal or >11 years	28	52.8	17	50	
**Surgery for IBD**	17	32.1	13	38.2	0.5553
**Active IBD at ophthalmologic outpatient visit**	14	26.4	9	26.5	0.9954
**IBD type**					0.2996
CD	35	66	26	76.5	
UC	18	34	8	23.5	
**IMIDs (APS, RA, JA and/or Psoriasis)**	22	41.5	12	35.3	0.5621
**Other EIM**	21	39.6	13	38.2	0.897
**IBD Treatment**					
Steroids	0	0	1	2.9	0.3908
5 amino-salicylates	7	13.2	2	5.9	0.4727
Immunosuppressants	7	13.2	2	5.9	0.4727
Other biologics	2	3.8	4	11.8	0.1512
Anti TNF	28	52.8	17	50	0.8289

* Chi-2 or Fisher’s exact test for qualitative variables, test from a Student or Wilcoxon test for quantitative variables. APS: ankylosing spondylitis, CD: Crohn’s disease; EIM(s): extra-intestinal manifestation(s); IBD: inflammatory bowel disease; IMID(s): immune-mediated inflammatory disease(s); JA: juvenile arthritis; RA: rheumatoid arthritis; TNF: tumor necrosis factor; UC: ulcerative colitis.

**Table 4:** Risk factors for ocular manifestations in patients with Crohn’s disease.

	Related to IBD		Not Related to IBD		
	*n* = 35 (57.4%)		*n* = 26 (42.6%)		
Characteristics	*n*	%	*n*	%	*p* *
**Gender**					0.5226
Male	12	34.3	11	42.3	
Female	23	65.7	15	57.7	
**Smoking**					0.1722
Active smoker	9	25,7	11	42.3	
Non-smoker or former smoker	26	74.3	15	57.7	
**Median age at IBD diagnosis (years [Q1; Q3])**	35	35 [23; 42]	26	26 [19; 43]	0.5046
**Median age at ophthalmologic outpatient visit (years [Q1; Q3])**	35	45 [35; 54]	26	47.5 [34; 59]	0.6135
**Date of ophthalmologic visit in comparison with IBD diagnosis**					NR
Before	2	5.7	0	0	
After	32	91.4	26	100	
Concomitant	1	2.9	0	0	
**Median time from IBD diagnosis to ophthalmologic outpatient visit (years)**					0.6273
<11 years	18	51.4	15	57.7	
Equal or >11 years	17	48.6	11	42.3	
**Surgery for IBD**	16	45.7	12	46.2	0.9728
**Active IBD at ophthalmologic outpatient visit**	9	25.7	4	15.4	0.7153
**IMIDs (APS, RA, JA and/or Psoriasis)**	16	45.7	11	42.3	0.7911
**Other EIM**	14	40	12	46.2	0.794
**IBD Treatment**					
Steroids	0	0	1	3.8	NR
Salicylates	1	2.9	0	0	NR
Immunosuppressants	5	14.3	1	3.8	0.2269
Other biologics	1	2.9	2	7.7	NR
Anti TNF	19	54.3	14	53.8	0.9728
**Montreal classification L**					0.5339
L1	11	31.4	7	26.9	
L2	10	28.6	5	19.2	
L3	14	40	14	53.8	
L4					NR
**Montreal classification B**					0.3706
B1	26	74.3	15	57.7	
B2	5	14.3	5	19.2	
B3	4	11.4	6	23.1	
**Perianal disease**	9	25.7	4	15.4	0.3299

* Chi-2 or Fisher’s exact test for qualitative variables, test from a Student or Wilcoxon test for quantitative variables. NR: not feasible due to low numbers in one of the modalities (less than 5). APS: ankylosing spondylitis, CD: Crohn’s disease; EIM(s): extra-intestinal manifestation(s); IBD: inflammatory bowel disease; IMID(s): immune-mediated inflammatory disease(s); JA: juvenile arthritis; RA: rheumatoid arthritis; TNF: tumor necrosis factor.

**Table 5:** Risk factors for ocular manifestations in patients with ulcerative colitis.

	Related to IBD		Not Related to IBD		
	*n* = 18 (69.2%)		*n* = 8 (30.8%)		
Characteristics	*n*	%	*n*	%	*p* *
**Gender**					0.6828
Male	7	38.9	4	50	
Female	11	61.1	4	50	
**Smoking**					0.3301
Active smoker	3	16.7	3	37.5	
Non-smoker or former smoker	15	83.3	5	62.5	
**Median age at IBD diagnosis (years [Q1; Q3])**	18	30 [23; 42]	8	31.5 [21.5; 50]	0.7414
**Median age at ophthalmologic outpatient visit (years [Q1; Q3])**	18	47.5 [35; 53]	8	46.5 [42.5; 63]	0.4432
**Date of ophthalmologic outpatient visit in comparison with IBD diagnosis**					NR
Before	1	5.6	0	0	
After	17	94.4	8	100	
**Median time from IBD diagnosis to ophthalmologic outpatient visit (years)**					0.6673
<11 years	7	38.9	2	25	
Equal or >11 years	11	61.1	6	75	
**Surgery for IBD**	1	5.6	1	12.5	NR
**Active IBD at ophthalmologic visit**	6	33.3	2	25	1
**IMIDs (APS, RA, JA and/or Psoriasis)**	6	33.3	1	12.5	0.3748
**Other EIM**	7	38.9	1	12.5	0.3602
**IBD Treatment**					
Steroids	0	0	0	0	NR
5 amino-salicylates	6	33.3	2	25	1
Immunosuppressants	2	11.1	1	12.5	NR
Other biologics	1	5.6	2	25	NR
Anti TNF	9	50	3	37.5	0.6828
**Montreal classification E**					0.5814
E1	6	33.3	3	37.5	
E2	6	33.3	1	12.5	
E3	6	33.3	4	50	

* Chi-2 or Fisher’s exact test for qualitative variables, test from a Student or Wilcoxon test for quantitative variables. NR: not feasible due to low numbers in one of the modalities (less than 5). EIM(s): extra-intestinal manifestation(s); IBD: inflammatory bowel disease; IMID(s): immune-mediated inflammatory disease(s); JA: juvenile arthritis; RA: rheumatoid arthritis; TNF: tumor necrosis factor; UC: ulcerative colitis.

## Data Availability

The data underlying this article are available in the article.

## Supplementary Materials

The following supporting information can be downloaded at: https://www.mdpi.com/article/10.3390/jcm11154538/s1. Figure S1. Ophthalmological diagnosis and supposed link with IBD.

## Abbreviations

AMD (Age-related Macular Degeneration), CD (Crohn’s Disease), CRAO (Central Retinal Artery Occlusion), CRSC (Central Serous Chorioretinopathy), ECCO (European Crohn’s and Colitis Organization), EIM (s) (Extra-Intestinal Manifestation(s)), HBI (Harvey-Bradshaw Index), IBD (Inflammatory Bowel Disease), IMID (s) (Immune-Mediated Inflammatory Disease(s)), JA (Juvenile Arthritis), PUK (Peripheral Ulcerative Keratitis), RA (Rheumatoid Arthritis), TNF (Tumor Necrosis Factor), UC (Ulcerative Colitis)
