# Supplementary figures and images for: Ocular Manifestations in Patients with Inflammatory Bowel Disease in the Biologics Era
Source: J Clin Med. 2022 Aug 4;11(15):4538. doi: 10.3390/jcm11154538 (PMC9369806; doi:10.3390/jcm11154538)

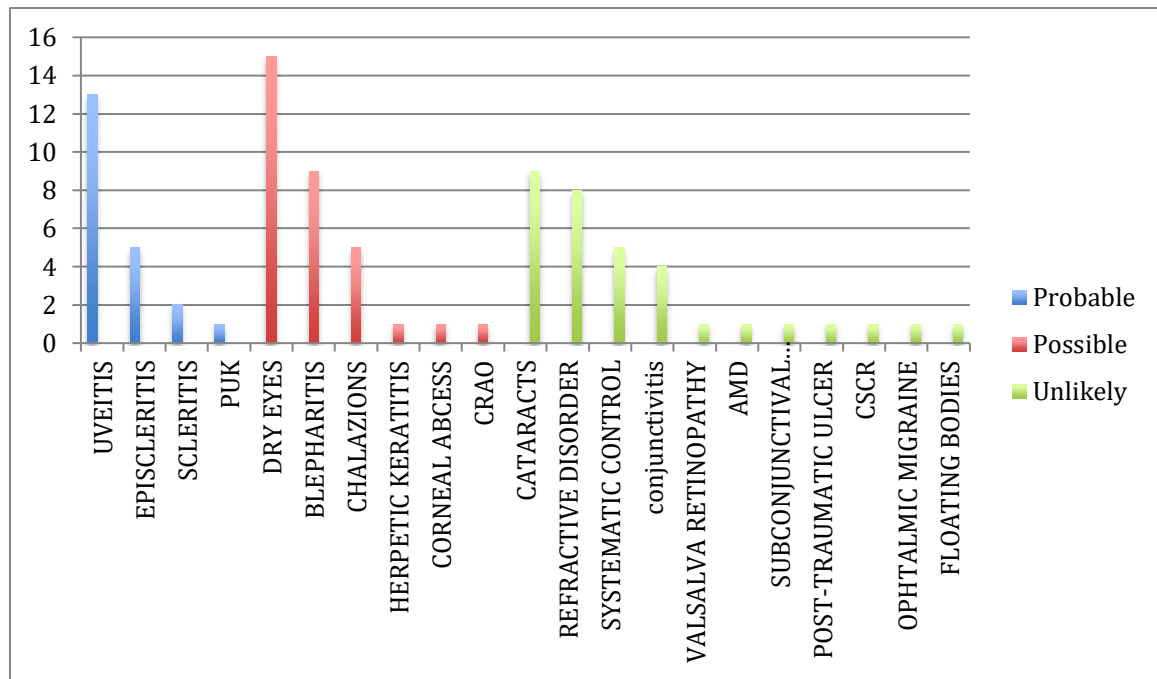

Supplementary Figure S1.

Supplement: Supplementary file 1 [file jcm-11-04538-s001.zip › jcm-1792947-supplementary.pdf]
